# Sensitive Detection of Chromosomal Segments of Distinct Ancestry in Admixed Populations

**DOI:** 10.1371/journal.pgen.1000519

**Published:** 2009-06-19

**Authors:** Alkes L. Price, Arti Tandon, Nick Patterson, Kathleen C. Barnes, Nicholas Rafaels, Ingo Ruczinski, Terri H. Beaty, Rasika Mathias, David Reich, Simon Myers

**Affiliations:** 1 Department of Epidemiology, Harvard School of Public Health, Boston, Massachusetts, United States of America; 2 Department of Biostatistics, Harvard School of Public Health, Boston, Massachusetts, United States of America; 3 Broad Institute of Harvard and MIT, Cambridge, Massachusetts, United States of America; 4 Department of Genetics, Harvard Medical School, Boston, Massachusetts, United States of America; 5 Johns Hopkins Allergy and Asthma Center, Division of Clinical Immunology, Department of Medicine, School of Medicine, Baltimore, Maryland, United States of America; 6 Department of Biostatistics, Johns Hopkins School of Public Health, Baltimore, Maryland, United States of America; 7 Inherited Disease Research Branch, National Human Genome Research Institute, National Institutes of Health, Baltimore, Maryland, United States of America; 8 Department of Statistics, Oxford University, Oxford, United Kingdom; 9 Wellcome Trust Centre for Human Genetics, University of Oxford, Oxford, United Kingdom; University of Chicago, United States of America

## Abstract

Identifying the ancestry of chromosomal segments of distinct ancestry has a wide range of applications from disease mapping to learning about history. Most methods require the use of unlinked markers; but, using all markers from genome-wide scanning arrays, it should in principle be possible to infer the ancestry of even very small segments with exquisite accuracy. We describe a method, HAPMIX, which employs an explicit population genetic model to perform such local ancestry inference based on fine-scale variation data. We show that HAPMIX outperforms other methods, and we explore its utility for inferring ancestry, learning about ancestral populations, and inferring dates of admixture. We validate the method empirically by applying it to populations that have experienced recent and ancient admixture: 935 African Americans from the United States and 29 Mozabites from North Africa. HAPMIX will be of particular utility for mapping disease genes in recently admixed populations, as its accurate estimates of local ancestry permit admixture and case-control association signals to be combined, enabling more powerful tests of association than with either signal alone.

## Author Summary

The genomes of individuals from admixed populations consist of chromosomal segments of distinct ancestry. For example, the genomes of African American individuals contain segments of both African and European ancestry, so that a specific location in the genome may inherit 0, 1, or 2 copies of European ancestry. Inferring an individual's local ancestry, their number of copies of each ancestry at each location in the genome, has important applications in disease mapping and in understanding human history. Here we describe HAPMIX, a method that analyzes data from dense genotyping chips to infer local ancestry with very high precision. An important feature of HAPMIX is that it makes use of data from haplotypes (blocks of nearby markers), which are more informative for ancestry than individual markers. Our simulations demonstrate the utility of HAPMIX for local ancestry inference, and empirical applications to African American and Mozabite data sets uncover important aspects of the history of these populations.

## Introduction

The identification of chromosomal segments of distinct continental ancestry in admixed populations is an important problem, with a wide range of applications from disease mapping to understanding human history. Early efforts to solve this problem used coarse sets of unlinked markers [1]–[3] and mostly focused on populations such as African Americans [4],[5] and Latinos [6]–[8] that admixed within the past approximately 10 generations. Applying this approach to more anciently admixed populations has led to ancestry predictions that are ambiguous at many loci [9]. However, methods based on coarse sets of markers do not take advantage of the much richer haplotype information available in genome-wide data. More recent methods have been designed to use data from genome-wide scanning arrays [10]–[12], but these methods do not fully model linkage disequilibrium (LD) in the ancestral populations. Thus, they do not capture all of the available information about ancestry, and can be far from optimal. Furthermore, unless a trimming step is applied to remove linked markers [11], unmodeled LD may cause systematic biases in estimated ancestry, leading to false-positive inferences of a deviation in ancestry at certain loci [13].

Here, we describe a haplotype-based method, HAPMIX, which applies an extension of the population genetic model of Li and Stephens [14] to the problem of local ancestry inference in populations formed by two way admixture. We apply the method to simulated mixtures of African and European chromosomes to show that the resulting local ancestry inference is exceedingly accurate in comparison to other methods, even in the case of ancient admixture in which the shorter ancestry segments are more difficult to infer. As expected from its use of an explicit population genetic model, HAPMIX makes more complete use of dense genome-wide data, producing more accurate results. We examine the sensitivity of local ancestry inference to a wide array of factors. We also explore the utility of HAPMIX for drawing inferences about both the ancestral populations and the date of admixture.

We apply HAPMIX to 935 African American individuals genotyped at ∼650,000 markers. By studying a large set of individuals from an admixed population of high relevance to disease mapping, we validate the effectiveness of this method in a practical setting and specifically show that the ancestry estimates are not systematically biased within the limits of our resolution. To illustrate how the method can provide insights into the history of an anciently admixed population, we also apply HAPMIX to a data set of 29 individuals from the Mozabite population of northern Africa that were genotyped at ∼650,000 markers as part of the Human Genome Diversity Panel (HGDP) [15]. We show that the Mozabite have inherited roughly 78% ancestry from a European-related population and 22% ancestry from a population related to sub-Saharan Africans. Our analysis also shows that the Mozabite admixture has occurred over a period that began at least 100 generations ago (∼2,800 years ago), and that has continued into the present day. We are able to infer small, ancient, ancestry segments in the Mozabite, and we demonstrate that the segments show considerable drift relative to all the other HGDP populations, consistent with the historical isolation of the Mozabite population.

## Materials and Methods

### Ethics statement

For the African American data, informed consent was obtained from each study participant, and the study protocol was approved by the institutional review board at either the Johns Hopkins University or Howard University.

### Overview of haplotype-based inference of local ancestry

HAPMIX assumes that the admixed population being analyzed has arisen from the admixture of two ancestral populations, and that phased data are available from unadmixed reference populations that are closely related to the true ancestral populations (e.g. phased data from HapMap [16]). In theory, discrepancies between the reference populations and the true ancestral populations may lead to inaccuracies, but in practice HAPMIX is robust to this concern under a variety of realistic scenarios (see below).

**Figure 1:**
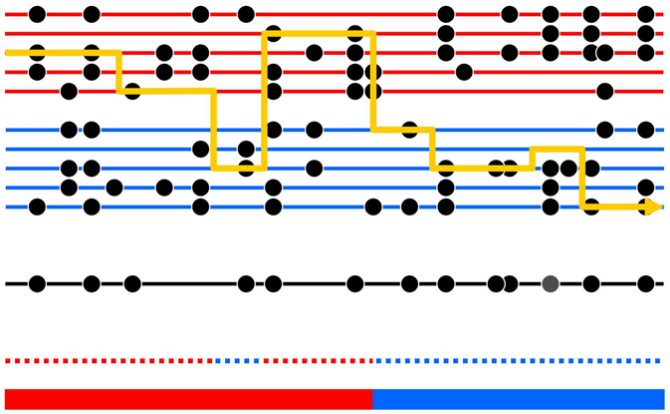
Schematic of the Markov model we use for ancestry inference. The black lower line represents a chromosomal segment from an admixed individual, carrying a number of typed mutations (black circles). The underlying ancestry is shown in the bottom color bar, and reveals an ancestry change from the first population (red) to the second population (blue). The admixed chromosome is modeled as a mosaic of segments of DNA from two sets of individuals drawn from different reference populations (red and blue horizontal lines respectively) closely related to the progenitor populations for the admixture event. The yellow line shows how the admixed chromosome is constructed in terms of this mosaic. The dotted line above the bottom color bar shows the reference population being copied from along the chromosome – note that at most positions, this is identical to the true underlying ancestry, but with occasional “miscopying” from the other population (blue dotted segment occurring within red ancestry segment). Note also that switches between chromosomes being copied from, representing historical recombinations, are rapid (6 switches), while ancestry changes, representing recombination since admixture, are much rarer (1 switch). Finally, note that at most positions the type of the admixed chromosome is identical to that of the chromosome being copied from, but an exception to this occurs at one site, shown as a grey circle, and representing mutation or genotyping error. In our inference framework, we observe only the variation data for the admixed and reference individuals: the yellow line, and the underlying ancestry, must be inferred as the hidden states in a HMM.

The central idea of the method is to view haplotypes of each admixed individual as being sampled from the reference populations: for example, haplotypes of an African American individual could be sampled from phased African and European chromosomes from HapMap. At each position in the genome, HAPMIX estimates the likelihood that a haplotype from an admixed individual is a better statistical match to one reference population or the other. A Hidden Markov Model (HMM) is used to combine these likelihoods with information from neighboring loci, to provide a probabilistic estimate of ancestry at each locus. The method allows transition at two scales. The small-scale transitions are between haplotypes from within a reference population, typically at a scale of every few tens of thousands of bases [14]. The large-scale transitions are between the reference populations, at a scale of up to tens of millions of bases for a recently admixed population such as African Americans. Figure 1 illustrates the method schematically.

An important strength of HAPMIX is the way it analyzes diploid data from admixed individuals. A naïve way to use population genetic methods to infer ancestry would be to pre-process such a data set using phasing software, and then to assume that this guess about the underlying phased haplotype is correct. However, phase switch errors that arise from this procedure (which are common even with the best phasing algorithms [17],[18]) would inappropriately force the method to infer ancestry transitions. HAPMIX circumvents this problem by not assuming that any one haplotype phase solution is correct. Instead, it uses a built-in phasing algorithm, similar to that of [17], which allows it to average inferences about ancestry over all possible phase solutions within each admixed individual. We treat the reference populations as fully phased, partly because in some cases, e.g. African and European chromosomes from HapMap, this phasing uses unambiguous trio information and is therefore highly accurate. More importantly, we expect our approach to be robust to errors in phasing in the reference populations, because these are unlikely to force inappropriate ancestry switches, in contrast to phasing errors in the admixed data itself.

HAPMIX is also notable in inferring probabilities for whether an individual has 0, 1, or 2 alleles of a particular ancestry at each locus. As our simulations show, these estimates are well-calibrated. Thus, when the method generates a probability *p* for an individual being heterozygous for ancestry at a locus, they are in fact heterozygous approximately this proportion of the time. A well-calibrated probability of ancestry at each locus is important for a variety of applications, and also allows us to evaluate the robustness of the results.

HAPMIX is fundamentally different from existing methods such as ANCESTRYMAP and LAMP [1],[11]. ANCESTRYMAP applies a Hidden Markov Model to unlinked SNPs to model ancestry transitions, while LAMP computes a majority vote of ancestry information using windows of unlinked SNPs, but neither of those methods makes use of haplotype information. Another method for investigating admixture segments, HAPAA, has recently been published [19]. In common with HAPMIX, the HAPAA software uses a Hidden Markov Model to model linkage disequilibrium within populations, and infers ancestry segments. However, there are also a number of important differences between our model and that used by HAPAA. First, unlike HAPAA, we allow for some rate of miscopying of ancestry segments from the “wrong” population, which we have found greatly improves our ancestry estimation (instead of this, the HAPAA software uses a post-hoc “filtering” of inferred segments, which removes all segments of size below a certain minimum threshold). Second, we fully allow for unphased data in our model, while the HAPAA approach requires a prior phasing of the data, and then attempts to account for the effect of phase-flip errors on ancestry inference via a heuristic procedure. We believe that these features of HAPMIX are likely to be critical in unraveling older admixture events, where ancestry segments are much shorter. A final advantage of HAPMIX over HAPAA is that it is designed to produce accurate estimates of uncertainty in inferred segments, even for old admixture events.

### Details of haplotype-based inference of local ancestry

#### Modeling genetic variation in admixed populations

Our approach to inferring ancestry segments, implemented in HAPMIX, is based on extending a Hidden Markov Model (HMM) previously developed by Li and Stephens to model linkage disequilibrium in population genetic data [14]. This model has been employed in recent years in various population genetic and disease mapping settings [20],[21]. Informally, given a previous collection of “parental” haplotypes from a reference population, a new “offspring” haplotype drawn from the same population is modeled as a mosaic of these existing haplotypes. This offers a flexible means to account for local linkage disequilibrium (LD), because over short distances, the haplotype that an individual chromosome copies from is unlikely to change.

We extend the Li and Stephens model to allow inference on ancestry segments for individuals drawn from an admixed population. We begin by supposing that we have two previously sampled collections of phased haplotypes, *P*_1_ and *P*_2_, taken from two reference populations. For example, HapMap provides phased haplotypes from the CEU, YRI and JPT+CHB populations genotyped at over 3 million markers [16]. We further assume that *P*_1_ and *P*_2_ have valid data at all sites of interest, with no missing data. In practice, small amounts of missing data in the reference populations can be filled in by a pre-processing imputation step, as has been done for the publicly available phased HapMap data. We label *P*_1_ and *P*_2_ as “parental” haplotypes. Next, we sample a new “offspring” haplotype from an admixed population. We assume that this population is created from a single admixture event between two populations which are genetically similar to the two reference populations from which *P*_1_ and *P*_2_ are drawn. (The reference populations do not need to *exactly* match the true ancestral populations, because we allow for some genetic divergence in our approach.) We will initially consider the case where we have haploid chromosomes from the admixed population, and subsequently generalize to the more typical case involving unphased genotype data from the admixed population. Throughout this section, we operate in units of genetic (not physical) distance.

We begin by modeling the ancestry segments. Assume the admixture event occurred at a single time *T* generations ago, with a fraction *μ*_1_ of the haplotype's ancestry drawn from population 1, and *μ*_2_ = 1−*μ*_1_ from population 2. Because recombination occurs at each generation, it is natural to model ancestry switches as a Poisson process along the genome [22], at a rate *T* per unit of genetic distance (i.e. *T* per Morgan). Conditional on the positions of such switches, each segment is independently drawn from population 1 or 2 with probabilities *μ*_1_, *μ*_2_ respectively. In particular, this implies that not all ancestry switch points will actually change the underlying ancestry. This model has been previously used by other authors [1],[22]. Since ancestry cannot be directly observed, it is natural to view underlying ancestry status as the “hidden” information in an HMM. Our approach probabilistically infers this hidden state at each position along a chromosome.

To fully specify our model, we must consider the structure of variation conditional on these admixture segments. Our model remains computationally tractable while accommodating important features typical of real data such as mutation, recombination, genotyping error, reference populations that are drifted from the true ancestral populations, and incomplete sampling of diversity in the reference populations reflected in the samples drawn from these populations. We assume that all mutant sites take the form of single nucleotide polymorphisms (SNPs) with two alleles that can be represented as 0 and 1 (however, our approach could be extended to more complex mutation models).

We suppose that sections of the genome with true ancestry from population 1 are formed as mosaics of the haplotypes in the two parental groups. Specifically, at any given position with this ancestry, an individual from *P*_1_ is copied with probability, and an individual from population *P*_2_ is copied with probability 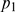 (we call this the “miscopying” parameter for population 1). Conditional on the parental group chosen, individuals to copy from are chosen uniformly from the *n*_1_, *n*_2_ respective individuals in that group. Switches between individuals occur as a Poisson process with rate *ρ*_1_, the “recombination” parameter, and at each switch point a new copy individual is chosen randomly using the above scheme. Finally, at genotyped SNPs, if the “offspring” copies a “parent” 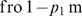 population 1, the offspring carries an identical type to the particular parent it copies from with probability (1−*θ*_1_), and carries the other type with probability *θ*_1_, the “mutation” parameter. If the offspring instead copies an individual from the other population 2, the corresponding mutation parameter is *θ*_3_. In total this approach leads to 4 additional parameters: *p*_1_, *ρ*_1_, *θ*_1_ and *θ*_3_.

For sections of the genome with ancestry from population 2, we formulate our model in an analogous way, with corresponding parameters *p*_2_, *ρ*_2_, *θ*_2_ and *θ*_3_. We note that *θ*_3_ is shared for both populations, a choice that is motivated by a genealogical argument, and has the aim of keeping the total number of parameters manageable. In total, our model has 9 independent parameters: *T*, *μ*_1_, *p*_1_, *p*_2_, *ρ*_1_, *ρ*_2_, *θ*_1_, *θ*_2_ and *θ*_3_.

Some additional remarks about the interpretation of these parameters may be useful. As in the original Li and Stephens implementation, *ρ*_1_ and *ρ*_2_ relate to historical recombination parameters. In our parameterization, these parameters depend on both the effective population sizes of the relevant populations, and the sample sizes *n*_1_ and *n*_2_ drawn from these populations. Although they are not merely a simple function of these quantities, informal coalescent-based arguments suggest that they will decrease roughly linearly with *n*_1_ and *n*_2_, and increase roughly linearly with the effective population sizes of the reference populations [14]. In general, because the amount of historical recombination depends on effective population size, we do not expect *ρ*_1_ = *ρ*_2_, even if *n*_1_ = *n*_2_. The mutation parameters *θ*_1_, *θ*_2_ and *θ*_3_ allow for both historical mutation and genotyping error. The miscopying parameters *p*_1_ and *p*_2_ allow similar “fuzziness” in the group copied from within ancestry segments. If 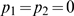, ancestry segments corresponding to population 1 must copy individuals from population 1, and similarly for population 2. However, setting these parameters equal to zero is likely to lead to spurious ancestry breaks, and therefore misestimation of ancestry segments, for at least two reasons. First, because we only sample a finite number of parental chromosomes, incomplete lineage sorting can occur. In some parts of the genome, the offspring chromosome is expected to have a deep coalescence time with the ancestors of the “correct” parental sample, and may instead coalesce first with an ancestor of the other parental sample – and therefore choose a descendant of this ancestor, in the “wrong” parental sample, to copy from. Second, if our reference populations are somewhat inaccurate relative to the true ancestral populations, again it is likely that incomplete lineage sorting will occur, even if our “parental” samples are both large. For these reasons, in practice we believe that incorporating non-zero miscopying parameters is important, and in both real data and simulation we find that it greatly improves our ancestry estimation procedure. Because our miscopying parameter is designed to allow for regions in the genome where the offspring chromosome has an unusually deep coalescence time with the other sample members, allowing the “miscopying” to occur, miscopied regions are likely to have unusually deep genealogies. Therefore, we allow a different mutation rate *θ*_3_ for such segments, which is typically expected to be higher than *θ*_1_ or *θ*_2_. It might also be desirable to allow a higher recombination rate in such cases. However, this would result in computational complexities, and we have chosen not to allow such an additional parameter.

For a typical application of HAPMIX, we expect to have data from a collection of discrete typed sites. Suppose we have *S* such sites, and in addition a map giving the genetic distances *r_1_*,*r_2_*,*…r_(S−1)_* between adjacent pairs of sites. In practice, we interpolate these distances from the genome-wide recombination rates estimated using Phase II HapMap [16]. Given the above parameters, and for a haploid admixed chromosome, we formalize the transition probabilities as follows. A (hidden) state for position *s* is represented by a triplet (*i*,*j*,*k*) where *i* = 1 or 2 represents ancestry drawn from population 1 or population 2, *j* = 1 or 2 records the population the chromosome copies from at position *s* (*j* may be different from *i* due to miscopying) and *k* represents the individual from which the chromosomal segment is copied. There are 2(*n_1_*+*n_2_*) possible states. Let 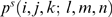 be the probability of transitioning from state (*i*,*j*,*k*) to state (*l*,*m*,*n*) between adjacent sites *s* and (*s*+1). Then we have the following:
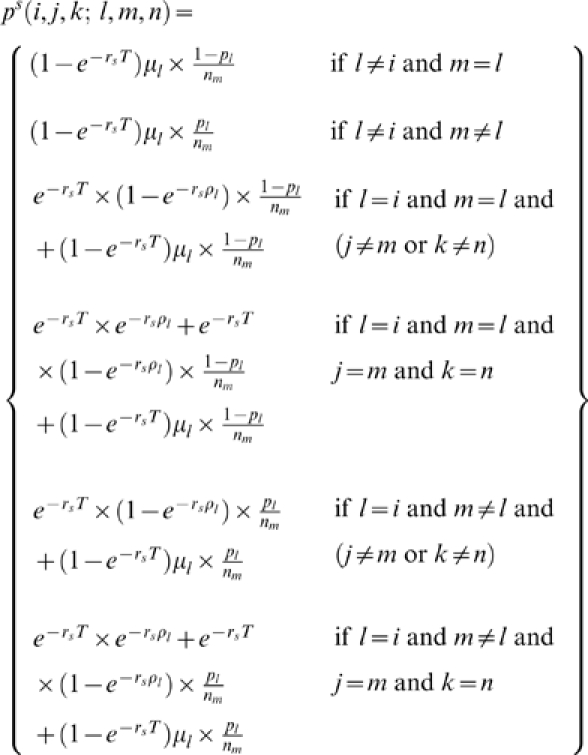
(0.1)Conditional on the underlying hidden state, let 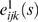 denote the probability of the offspring chromosome being of type 1 at site *s*, and *t_jk_* be the type of parental individual *k* in reference population *j*. Then
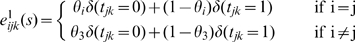
(0.2)


This probability allows us to calculate the likelihood of the observed data in the offspring for each possible underlying state. At sites with missing data in the offspring chromosome, the appropriate likelihood contribution is simply 1.0.

#### Choices of parameter settings

Choices of *T* and *μ*_1_ are specific to each application (see below). However, many of the remaining parameters were fixed in all analyses of both simulated and real data. As discussed above, it is natural to scale *ρ*_1_ and *ρ*_2_, as well as *θ*_1_ and *θ*_2_, by the numbers of parental individuals *n_1_*, *n_2_*, respectively. Our code is parameterized so this is done internally – arbitrarily labeling the European population as ancestral population 1, we used recombination parameters *ρ*_1_ = 60,000/*n_1_* per Morgan for the European ancestral population and *ρ*_2_ = 90,000/*n_2_* per Morgan for the African ancestral population (with *ρ*_2_>*ρ*_1_ reflecting the larger effective population size of Africans). Further, we set *θ*_1_ = 0.2/(0.2+*n_1_*) and *θ*_2_ = 0.2/(0.2+*n_2_*), and *θ*_3_ = 0.01 (this parameter remains unscaled). Finally, we used miscopying parameters *p*_1_ = *p*_2_ = 0.05. These values were arrived at via a process of trial and error, based on the results of inferring parameters via the EM algorithm. We have implemented an EM algorithm approach to parameter estimation that can infer any subset of the HAPMIX input parameters, or all simulataneously (see Text S2). This EM approach to parameter inference is currently only implemented for haploid data from the admixed population, but we applied it to haploid data derived from a phasing of diploid data, obtained by running HAPMIX on diploid admixed samples and using the software to sample random state paths. This approach might be applied to diploid samples more generally, and could be potentially be iterated, by updating phasing based on new parameter sets. However, based on our simulations we believe that for many applications – for example whenever the software is applied to African American data - it will be sufficient to vary *T* and *μ*_1_ and fix the remaining parameters at the values described above.

#### Inferring probabilistic ancestry segments and sampling from the posterior with HAPMIX

It is easy to see that equations (0.1) and (0.2) describe a HMM for the underlying state (which includes information on ancestry) as we move along the genome, and that the underlying Markov process is reversible. Given a set of parameters we can exploit these properties and HAPMIX implements standard HMM techniques to efficiently infer posterior probabilities of underlying states, via the forward-backward algorithm, or sample random state paths from the correct joint posterior distribution, using a standard modification of this algorithm. In addition to parameter values, the software takes as input a recombination map for the regions to be analyzed, phased “parental” chromosomes from the two reference populations, and “offspring” data from the admixed population being analyzed.

A naïve implementation of the forward/backward algorithm would require computation time proportional to 4*S*(*n_1_*+*n_2_*)^2^, in the above notation. For the original Li and Stephens model, it is possible to reduce computation time substantially by using the fact that many pairs of transition probabilities between states are identical, which allows terms to be collapsed in the forward (or backward) algorithm, into expressions involving a single term that is shared among all destination states. Calculating this shared term just once per pair of adjacent sites, and then storing, saves substantial computational effort [14]. Analogously, in our somewhat more complicated setting we can exploit a similar phenomenon, so that by calculating and storing a somewhat larger number of shared terms – one for each group of states of the form (*i*,*j*), giving four in total - HAPMIX can complete the forward/backward algorithm in time proportional to 2*S*(*n_1_*+*n_2_*) (with an additional scaling constant).

It is straightforward to extend our approach to allow imputation of missing data, while simultaneously labeling underlying ancestry, in an analogous manner to methods employed in several existing approaches to imputation for samples drawn from panmictic populations [20],[21]. We will describe this extension, and its application to disease mapping, in a separate paper.

#### Multiple individuals from the admixed population

Typically, we actually have multiple “offspring” samples (either haploid chromosomes or diploid genotypes, see below) from the admixed population of interest. For the analyses in this paper, we used HAPMIX to analyze data from each sample independently, using the same parental chromosomes in each case. Although in principle improvements to ancestry inference could result from considering the problem in multiple samples jointly, there are formidable computational challenges in adapting our approach to allow this (one possibility might be to employ MCMC, as used for unlinked sites [22],[23]). To avoid these complications, we simply model each admixed sample independently, following [21]. Under this scheme, separate HAPMIX runs for each sample enable effective parallelization of the software.

#### Diploid genotype data from the admixed population

Typically, real data consists of unphased genotypes for individuals drawn from a population, with haplotypic phase unknown. Many approaches already exist to infer phase from such data [17],[18]. However, phase switch errors that inevitably result from applying such algorithms are likely to result in spurious ancestry switches within regions of the genome where an individual is heterozygote for ancestry. This would likely lead to considerable overestimation of the time since admixture and a reduction in the accuracy of ancestral inference. To avoid such issues, we have extended our approach to directly analyze diploid genotype data from the admixed population.

The phasing is implemented using a HMM adapted from that described above (0.1) and employing a composite hidden state at each location, of the form (*i_1_*,*j_1_*,*k_1_*,*i_2_*,*j_2_*,*k_2_*) where (*i_1_*,*j_1_*,*k_1_*) represents the previously defined “haploid” hidden state for the first chromosome, and (*i_2_*,*j_2_*,*k_2_*) represents the hidden state for the second chromosome. The state space therefore now has dimension 4(*n*_1_+*n*_2_)^2^. Allowing independent transitions between the marginal states for each chromosome, the terms in (0.1) now naturally define an HMM for these composite states (for reasons of space, we do not explicitly list all of the transition probabilities in the model here). This model could have up to 18 parameters – in our implementation, for natural biological reasons we assume all parameters are shared between chromosomes, apart from time since admixture *T* and admixture proportion *μ*_1_, resulting in 11 parameters in total. Further, although our software allows these two parameters to differ, in all applications considered here we specify *T* and *μ*_1_ to be the same for each chromosome.

Emission probabilities are also adapted from the haploid case. For genotype data, there are 3 possible emissions at typed sites, which we denote as genotypes *g* = 0, 1, or 2, with *g* counting copies of the “1” allele. Conditional on the underlying hidden state, let 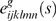 denote the probability of observing genotype *g* given underlying state (*i*,*j*,*k*,*l*,*m*,*n*), and define *t_jk_* as before to be the type of parental individual *k* in reference population *j*. Then using (0.2)
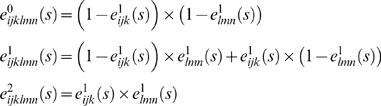
(0.3)where 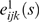 and 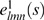 are as defined above.

Having defined the HMM for this setting, we again use standard techniques to obtain posterior probabilities on (joint) ancestry for the two chromosomes, and then sample states from this posterior distribution. We note that as a by-product of sampling complete states jointly for the two chromosomes together, we are phasing the original data with respect to the underlying ancestry. This may help reduce phasing error rates in admixed populations compared to methods that ignore local ancestry, although we do not pursue this issue here.

We can adapt the computational speedups described above to the diploid setting, so that while a naïve implementation of the forward algorithm would take time proportional to 16*S*(*n*_1_+*n*_2_)^4^, we can complete the forward/backward algorithm in time proportional to 4*S*(*n*_1_+*n*_2_)^2^. A further speedup for the diploid setting is described in Text S2. With these speedups implemented, the running time of HAPMIX is roughly 30 minutes on a single processor per diploid genome analyzed (519,248 sites). Because the computations can be parallelized across admixed individuals (they can also be parallelized across chromosomes), HAPMIX is computationally tractable even for very large data sets if a cluster of computing nodes is available. For example, the running time for a data set of 1,000 admixed individuals on a cluster of 100 nodes is roughly 5 hours.

### Measuring the performance of HAPMIX

#### Estimate of *r* ^2^ between predicted and true ancestry

Irrespective of whether the true ancestry is known (as in simulations) or unknown (as in real data), an estimate of the *r* ^2^ between a predicted ancestry vector **Y** and true ancestry **X** can be computed. Within an individual, at each site *s*, a natural measure of predicted ancestry is the expected number Y*_s_* of haplotypes from one of the two source populations. If HAPMIX provides accurate ancestry probabilities, the true number of haplotypes from this population, X*_s_*, can be thought of as an unknown random variable which is equal to 0, 1, or 2 with probabilities *p*_0_, *p*_1_, *p*_2_ specified by the ancestry predictions. We are interested in how correlated the predicted ancestry **Y** and true ancestry **X** are, over samplings from this distribution of the true ancestry **X**. A natural way to estimate this correlation is to calculate the expected squared correlation between **X** and **Y**, which we may approximate using a ratio of means:
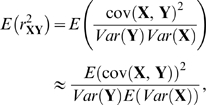
where the variances and covariances are taken over loci and individuals, and the expectations over samplings of the ancestry **X**. The expected covariance between predicted and true ancestry is then the mean value of the covariance between **X** and **Y** as we sample ancestry paths at different loci and in different individuals. At our single locus, we have E(*X_s_Y_s_*) = (*p*_1_+2*p*_2_)^2^ and E(*X_s_*) = E(*Y_s_*) = *p*_1_+2*p*_2_. By separately averaging these three expectations across loci and individuals, we can then calculate 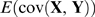 analytically. Similarly, we can calculate the variance of **Y**, and the expected variance of **X**, across loci and different individuals, in a similar way. Combining these variances with the covariance to estimate correlation, and then squaring, we obtain a measure of the level of certainty of the ancestry predictions.

#### Actual *r* ^2^ between predicted and true ancestry

In simulated data sets where the true ancestry is known, the estimated *r* ^2^ between predicted and true ancestry (which is computed using ancestry predictions only) can be compared to the actual *r* ^2^ between these quantities (comparing ancestry predictions to true ancestries specified in simulations). As we confirm in what follows, the estimates of *r* ^2^ are well calibrated.

### Simulations

#### Simulations of local ancestry inference

We simulated individuals of admixed African and European ancestry by constructing their genomes from a mosaic of real Yoruba and French individuals genotyped on the Illumina 650Y chip as part of the Human Genome Diversity Panel (HGDP) [15]. We downloaded data from 20 Yoruba and 20 French individuals from the HGDP data set and jointly phased them using the fastPHASE program [18] to form 40 haploid Yoruba and 40 haploid French genomes.

We constructed 40 haploid admixed genomes (*n* = 1 to 40) from the 40 haploid Yoruba and 40 haploid French genomes by using haploid Yoruba genome *n* and haploid French genome *n* to construct admixed genome *n*, so that ancestral genomes were never reused. To construct an admixed genome, we began at the first marker on each chromosome and sampled French ancestry with probability *α* and Yoruba ancestry with probability 1-*α*. Ancestry was resampled based on an exponential distribution with weight *λ* (the number of generations since admixture) so that a new ancestry was sampled with probability 1−e^−*λg*^ when traversing a genetic distance of *g* Morgans. Each time ancestry was resampled, we sampled French ancestry with probability *α* and Yoruba ancestry with probability 1-*α*. For each individual, we used a value of *α* to apply to the entire genome by sampling from a beta distribution with mean 0.20 and standard deviation 0.10 (typical for African Americans [4]). We simulated values of *λ* = 6 (typical for African Americans [4]) as well as higher values of *λ*: 10, 20, 40, 60, 100, 200 and 400. Pairs of haploid admixed individuals were merged to form 20 diploid admixed individuals.

It is important to distinguish between the *true* ancestry proportion *α* in a simulated or real admixed individual and the *parameter μ*_1_ used as input to HAPMIX, which may differ from *α* (if *α* is unknown). Similarly, it is important to distinguish between the *true* number *λ* of generations since admixture and the *parameter T* used as input to HAPMIX. Below we explore the consequences of inaccurately specifying the parameters *μ*_1_ and *T*.

The reference populations used as input to HAPMIX consisted of 60 YRI individuals (120 haploid chromosomes) and 60 CEU individuals (120 haploid chromosomes) from the International HapMap Project [16]. A joint analysis of HGDP and HapMap data indicated that *F*_ST_(Yoruba,YRI) = 0.000 and *F*_ST_(French,CEU) = 0.001, so that the reference populations used as input to HAPMIX were extremely accurate. All HAPMIX simulations were restricted to 519,248 autosomal markers present in HGDP data which were polymorphic in phased YRI and phased CEU data from HapMap. For comparison purposes, we ran the ANCESTRYMAP, and LAMP-ANC programs on the same simulated data sets, making use of diploid YRI and CEU genotype data from HapMap and restricting all input data to subsets of markers that were unlinked in the reference populations, as recommended by those methods [1],[11]. For the ANCESTRYMAP runs, we chose a subset of <8,000 markers with the largest YRI-CEU differences that were unlinked in both reference populations. For the LAMP-ANC runs, we set the LD cutoff to 0.10, causing the program to choose a subset of ∼260,000 markers. We note that LAMP-ANC differs from the LAMP program in that LAMP-ANC makes use of input data from reference populations [11], which makes it more comparable to HAPMIX. We attempted to run HAPAA on the same data for comparison purposes. However, despite advice from the authors of the software and extensive effort, we were unable to make the linked applications that form the HAPAA software suite run on our computers, and hence we were unable to make this comparison.

#### Simulations of local ancestry inference using inaccurate reference populations

We repeated our simulations at *λ* = 6 and *λ* = 100 using Mandenka from HGDP as the African ancestral population and Basque from HGDP as the European ancestral population for simulating admixed individuals. We simulated 20 admixed individuals using Mandenka and Basque data (analogous to the simulations described above using Yoruba and French data). We continued to use YRI and CEU as the reference populations for HAPMIX. A joint analysis of HGDP and HapMap data indicated *F*_ST_(Mandenka,YRI) = *F*_ST_(Basque,CEU) = 0.01. We note that these discrepancies between the ancestral populations used to construct these simulated data and the reference populations used as input to HAPMIX are substantially larger than the discrepancy between the true African ancestral population of African Americans and YRI, or the true European ancestral population of African Americans and CEU [4].

To investigate the scenario of an even more inaccurate reference population, as well as the asymmetric scenario in which only one reference population is inaccurate, we also repeated our simulations at *λ* = 6 and *λ* = 100 using Yoruba from HGDP as the African ancestral population and Druze from HGDP as the European ancestral population for simulating admixed individuals. We simulated 20 admixed individuals using Yoruba and Druze data as described above, and continued to use YRI and CEU as the reference populations. A joint analysis of HGDP and HapMap data indicated that *F*_ST_(Druze,CEU) = 0.02.

#### Simulations of local ancestry inference as a function of data size and parameter settings

We modified our original simulations at *λ* = 6 and *λ* = 100 to consider different data sizes and parameter settings. We investigated how the performance of HAPMIX varies as a function of data size by varying the number of markers from 5,192 randomly selected markers to the full set of 519,248 markers, and by varying the amount of input data from YRI and CEU reference populations from 10 haploid chromosomes to the full set of 120 haploid chromosomes. We investigated how the performance of HAPMIX varies as a function of parameter settings by incorrectly specifying either the European ancestry proportion *μ*_1_ used as input to HAPMIX (using values different from *α*≈20%) or the number of generations *T* since admixture used as input to HAPMIX (using values different from *λ* = 6 or *λ* = 100, respectively).

#### Inference of ancestral populations

By running HAPMIX in the mode that samples random paths, which produces integer-valued guesses of local ancestry for each individual and each marker, it is possible to reconstruct chromosomal segments from the ancestral populations. We investigated whether these reconstructed segments provide an accurate proxy for the true ancestral populations by using allele counts to compute values of *F*_ST_ (a standard measure of genetic distance [24]) between inferred ancestral segments and true ancestral populations from our simulations. Although ancestral individuals are used twice in this computation (both to simulate admixed individuals whose ancestral segments are inferred, and in the ancestral populations themselves), we restricted this analysis to half of the ancestral individuals for the former and the other half of the ancestral individuals for the latter, thus preventing any duplication of data in the computation of *F*_ST_. We performed this computation both for our original simulations in which the true ancestral populations (Yoruba and French) are accurately modeled by the reference populations used (YRI and CEU), and for our inaccurate ancestral population simulations in which true ancestral populations (either Mandenka and Basque, or Yoruba and Druze) are inaccurately modeled by the reference populations (YRI and CEU). We restricted these analyses to data simulated using *λ* = 6 and *λ* = 100 only.

#### Inference of date of admixture

By comparing the overall likelihoods produced by HAPMIX at various parameter settings, it is possible to evaluate which parameters provide the best fit to the data, irrespective of whether or not the choice of parameter settings significantly impacts the accuracy of local ancestry inference. We investigated how effectively the number of generations *λ* since admixture can be inferred in this way by running HAPMIX at various values of *T* and computing overall likelihoods, using the data sets simulated at *λ* = 6, *λ* = 20 and *λ* = 100. We also simulated a double-admixture scenario in which a 50%/50% admixture of Yoruba and French occurred at *λ* = 100 followed by a 50%/50% admixture of that population and French at *λ* = 6 (we call this the *λ* = 6∘100 run (with *α* = 75%)). We optimized *T* at a granularity of 1 for the *λ* = 6 and *λ* = 20 simulations and a granularity of 5 for the *λ* = 100 and *λ* = 6∘100 simulations.

### Analysis of 935 African American samples

We used HAPMIX to analyze 935 African American samples collected from volunteers living in the Baltimore–Washington, D.C. metropolitan region and genotyped on the Illumina 650Y chip as part of an asthma study. All subjects gave verbal and written consent. The Johns Hopkins and Howard University Institutional Review Boards (IRBs) determined that the samples were consented for genetic research, but not for public release of genotype data. Roughly half of these samples were asthma cases and half were non-asthmatic controls, but all phenotypic information was ignored in the current study (disease mapping analyses of these data will be described elsewhere; K. Barnes et al., unpublished data). We note that irrespective of whether asthmatic cases considered separately exhibit an admixture association signal, one would not expect to observe such a signal in a combined analysis of all 935 samples ignoring phenotypic information, due to dilution of the signal. The analyses were restricted to 510,324 autosomal markers which passed quality controls in the 935 African Americans and were polymorphic in phased YRI and phased CEU data from HapMap. We ran HAPMIX using YRI and CEU as input reference populations, setting *μ*_1_ = 20% and running at various values of *T* to infer the date of admixture (see above). For comparison purposes, we also ran the ANCESTRYMAP and LAMP-ANC programs on this data, in each case restricting all input data to a subset of markers that were unlinked in the reference populations, as described above.

To draw inferences about the ancestral populations of African Americans, we ran HAPMIX in the mode that samples random paths to reconstruct chromosomal segments from the ancestral populations (see above), and used the resulting allele counts to compute *F*_ST_ values between the inferred ancestral segments and the reference populations (YRI and CEU), as well as additional populations genotyped as part of the HGDP. To estimate the number of ancestry segment changes in each of the 935 African American individuals, we inferred ancestry using the most likely state at each site, and identified ancestry transitions from these ancestry states, assuming zero changes between pairs of SNPs with identical ancestry states.

To produce an estimator of the number of generations since admixture for each individual with >20 ancestry segments, we note that the genetic map used as input to the software has total length 35.5 Morgans. For an individual with admixture proportion α, we expect to observe a fraction 2α(1-α) of all recombination events occurring since admixture (i.e. those that result in a change in ancestry). Given λ generations since admixture, we therefore expect to see a total of 142 λ α(1-α) events in a diploid individual. Estimating α using the observed genome-wide ancestry proportion μ for that individual, if *N* ancestry transitions are observed, then a natural moment estimator of the number of generations since admixture is
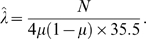



We excluded 3 clear outlier individuals who had more than 20 inferred generations of admixture, because we believe this is likely to indicate partial ancestry from a third source population in these individuals.

### Analysis of 29 Mozabite samples

We analyzed 29 Mozabite samples from the HGDP data set. A total of 30 Mozabite individuals were originally genotyped as part of the HGDP, but one individual (HGDP01281) was excluded due to cryptic relatedness. We ran HAPMIX on the 29 Mozabite individuals using YRI and CEU as the input reference populations. We inferred the number of generations since admixture that provided the best fit to the data, and computed *F*_ST_ values between the inferred ancestral segments and the reference populations (YRI and CEU), as described above for the African American data set.

### Analysis of other HGDP populations

We ran HAPMIX on a total of 13 populations from the HGDP data that were of African, European, or Middle Eastern ancestry. For each population, we used YRI and CEU as the input reference populations, and estimated the European-related mixture proportion. For populations with European-related ancestry that was estimated to be more than 0% and less than 100%, we also estimated the number of generations since mixture.

### Web resources

The HAPMIX software is available for downloading at the following URL: http://www.stats.ox.ac.uk/~myers/software.html.

## Results

### Simulations

#### Simulations of local ancestry inference

**Figure 2:**
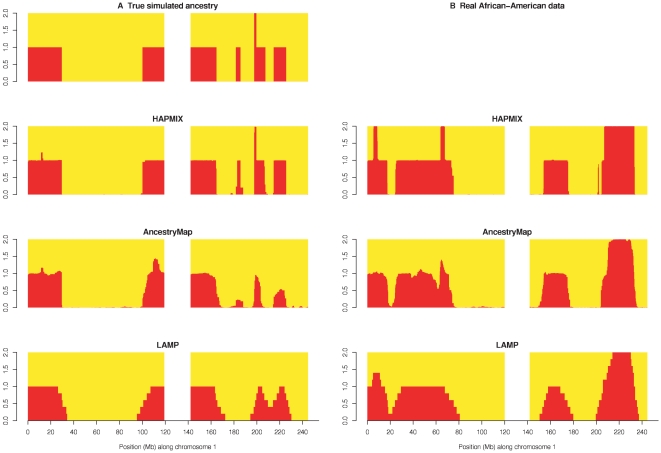
Comparison of ancestry estimates produced by HAPMIX, ANCESTRYMAP, and LAMP-ANC. (A) Results comparison for a simulated recently admixed sample on chromosome 1. On each plot, the y-axis denotes the number of European chromosomal copies predicted by each method. The centromere of the chromosome is blanked out in white. The top plot shows the true number of European chromosomes, while the subsequent labeled plots show the results of applying each respective method. (B) Results comparison for a real African American individual across chromosome 1. Plots are constructed as in (A). We note the visible similarity to the simulation results.

We began by examining the performance of HAPMIX in a set of 20 simulated admixed individuals, with an average of 80% African ancestry and 20% European ancestry, and generated with admixture occurring 6 generations ago (*λ* = 6; see Materials and Methods). These parameters were chosen to be in the range of typical values for African Americans. We implemented a simulation framework in which admixed individuals were constructed using genotype data from the Human Genome Diversity Project, but modeled using reference populations from HapMap (see Materials and Methods). We compared the local ancestry estimates produced by HAPMIX (probabilities of 0, 1, or 2 copies of European ancestry) to the true values of local ancestry that were simulated. These simulation results suggest that our method is likely to provide near optimal ancestry reconstruction in African Americans: the squared correlation between predicted and true number of European copies (across all samples) was equal to 0.98, and discernment of ancestry transitions was extremely sharp, as seen in a plot of the predicted vs. true number of European copies for an admixed sample on chromosome 1 (Figure 2A). For comparison purposes, we also computed local ancestry estimates using the ANCESTRYMAP and LAMP-ANC programs [1],[11] (see Materials and Methods). (We chose not to explicitly compare HAPMIX to additional recently developed methods such as SABER, LAMP, uSWITCH and uSWITCH-ANC [10]–[12] , because in previous work the LAMP-ANC method—which we do compare HAPMIX to—has been shown to perform approximately as well as each of those methods in a range of scenarios [11].) The squared correlation between predicted and true number of European copies was equal to 0.86 for ANCESTRYMAP, 0.83 for LAMP-ANC and discernment of ancestry transitions was less sharp or sometimes missed entirely (Figure 2A).

**Figure 3:**
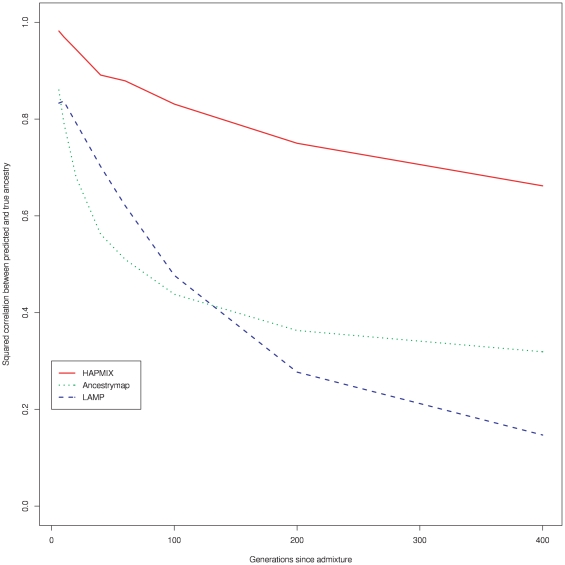
Accuracy of HAPMIX, ANCESTRYMAP, and LAMP-ANC predictions for various values of *λ*, the number of generations since admixture. For each admixture time, results are based on analyzing 20 admixed individuals, simulated using an average genome-wide proportion of 80% African and 20% European ancestry. For each method, we plot the squared correlation between predicted and true number of European copies as a function of *λ*.

A more challenging setting for ancestry inference is when admixture occurs further back in time, resulting in smaller ancestry segments. We therefore repeated the above comparisons with increasing lambda (Figure 3). The results show a uniformly better performance by HAPMIX relative to the other two methods, with the comparative advantage of HAPMIX increasing with time since admixture.

**Figure 4:**
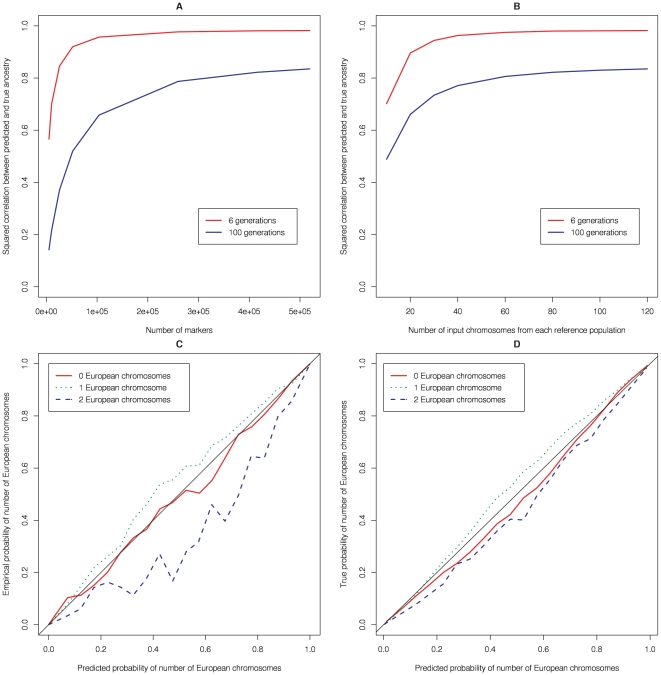
Properties of HAPMIX. (A) For simulated admixed data sets, constructed as described in Materials and Methods using *λ* = 6 and *λ* = 100, we plot the *r* ^2^ between predicted and true number of European chromosomal copies, as a function of the number of markers genotyped across the genome. (B) The same as part A, except we now fix the number of markers genotyped at 500,000, and vary the number of input chromosomes used to predict ancestry (for full details, see text). (C) Calibration of uncertainty estimates produced by HAPMIX. For the *λ* = 6 simulations, and for each of *x* = 0, *x* = 1, and *x* = 2 we compare the average probability of *x* copies of European ancestry predicted by HAPMIX to the true frequency of having *x* copies of European ancestry, binning the predicted probabilities of x copies of European ancestry into bins of size 0.05. If the method were perfectly calibrated, the results would lie along the line *y* = *x* (thin black line). Note that for *λ* = 6, ancestry is normally inferred with high certainty, and over 98% of data points fall into the most extreme two bins. (D) The same as part A, except using *λ* = 100. Both the last two plots show reasonable calibration of HAPMIX.

To investigate whether the probabilities of 0, 1, or 2 copies of European ancestry reported by HAPMIX are well-calibrated, we binned the predicted probabilities into bins of size 0.05 and compared, for each *x* = 0,1,2 and for each bin, the average predicted probability vs. the actual frequency in simulations of having *x* copies of European ancestry. For example, in the *λ* = 6 simulation, restricting to instances in which the predicted probability of 1 copy of European ancestry was between 0.05 and 0.10, the average predicted probability of 1 copy of European ancestry was 0.07 and the true frequency of 1 copy of European ancestry was 0.08, which is close to 0.07. More generally, we observed that HAPMIX predictions from our *λ* = 6 and *λ* = 100 simulations were well calibrated for each value of *x* = 0,1,2 (Figure 4). The calibration of intermediate bins appears visually worse for the *λ* = 6 simulation; however, the proportion of the genome that is in the most extreme bins where the method is certain is 98%, 97%, 99%, for x = 0,1,2 in these simulations, and hence the reliability of the probabilities remains good for recently admixed populations too.

We also used the HAPMIX predictions to compute an estimate of the squared correlation between predicted and true #European copies (see Materials and Methods). We obtained estimates of 0.98 for the *λ* = 6 simulation and 0.83 for the *λ* = 100 simulation, which are identical to the true *r* ^2^ values of 0.98 for *λ* = 6 and 0.83 for *λ* = 100, consistent with the finding that HAPMIX predictions are well calibrated.

Although most of our simulations focused on individuals of mixed African and European ancestry, we also considered a more general set of two-way mixtures of African, European, Chinese and/or Japanese populations. We again observed that HAPMIX outperformed other methods (see Text S1). Furthermore, although HAPMIX is currently implemented assuming only two reference populations, we were able to attain accurate results in a more complex scenario of three-way admixture, by running HAPMIX in a two-way mode using different choices of reference populations (see Text S1).

#### Simulations of local ancestry inference using inaccurate reference populations

In many real-world settings, the true reference populations for a particular admixture event may not have had suitable genetic data gathered, or may no longer exist. To test for the effect of this situation on HAPMIX, we repeated our simulations at *λ* = 6 and *λ* = 100 using 20 admixed samples that were simulated using Mandenka and Basque individuals but modeled using reference populations YRI and CEU, which are inaccurate reference populations (see Materials and Methods). For *λ* = 6, the squared correlation between predicted and true #European copies remained high at 0.95, only marginally worse than the 0.98 obtained using accurate reference populations. For *λ* = 100, the squared correlation was 0.76, again only slightly worse than the 0.83 obtained using accurate reference populations. In short, the effects of these levels of inaccuracy in the reference populations (*F*_ST_ = 0.01) are relatively small.

We also repeated our simulations at *λ* = 6 and *λ* = 100 using 20 admixed samples that were simulated using Yoruba and Druze but modeled using reference populations YRI and CEU, (see Materials and Methods). The squared correlation between predicted and true number of European copies was 0.97 at *λ* = 6 and 0.79 at *λ* = 100, as compared to 0.98 and 0.83 using accurate reference populations. Thus, HAPMIX is robust to rather inaccurate (*F*_ST_ = 0.02) reference populations, and to the asymmetric case where only one reference population is inaccurate.

#### Simulations of local ancestry inference as a function of data size and parameter settings

We investigated how the accuracy of HAPMIX varies with data size, by varying either the number of markers or the number of reference chromosomes, in our *λ* = 6 and *λ* = 100 simulations (see Materials and Methods). Accuracy as a function of the number of markers is displayed in Figure 4A, which shows that as few as 50,000 random markers are close to optimal for *λ* = 6 but that hundreds of thousands of markers are needed to produce optimal results in the more challenging case where *λ* = 100. Accuracy as a function of the number of reference chromosomes is displayed in Figure 4B, which shows that as few as 40 chromosomes (phased from 20 diploid samples) from each reference population are close to optimal.

We also investigated how the accuracy of HAPMIX is affected when the parameters used as input are inaccurately specified (see Materials and Methods). Results of our simulations in which the genome-wide ancestry proportion *μ*_1_ was inaccurately specified (different from the value *α* used to simulate the data) are displayed in Table 1. We observed that even if *μ*_1_ is very inaccurate (e.g. by a factor of 4), there is no effect on results for *λ* = 6 and only a minimal effect (which primarily affects the genome-wide average of HAPMIX ancestry estimates, but not their correlation with true ancestry) for *λ* = 100. Results of our simulations in which the number of generations *T* since admixture was inaccurately specified (different from the value *λ* used to simulate the data) are displayed in Table 2. We observed that even if *T* is very inaccurate (e.g. by a factor of 2 to 5), there is no effect on results for *λ* = 6 and only a minimal effect for *λ* = 100. Thus, HAPMIX appears to be extremely robust to parameter misspecification.

**Table 1:** HAPMIX accuracy as a function of ancestry proportion parameter.

*μ*_1_	*λ* = 6 simulated data: r^2^ (α_average_)	*λ* = 100 simulated data: r^2^ (α_average_)
0.05	0.98 (0.20)	0.82 (0.18)
0.10	0.98 (0.20)	0.83 (0.19)
0.20	0.98 (0.20)	0.83 (0.20)
0.40	0.98 (0.20)	0.83 (0.21)
0.80	0.98 (0.20)	0.83 (0.22)

We list both the *r* ^2^ between true and inferred ancestry, and the genome-wide average *α*_avg_ of HAPMIX ancestry estimates, as a function of the parameter *μ*_1_ used as input to HAPMIX, for data simulated at *λ* = 6 and *λ* = 100. Results for HAPMIX runs in which the ancestry proportion was specified correctly are underlined.

**Table 2:** HAPMIX accuracy as a function of date of admixture parameter.

*T*	*r* ^2^ for *λ* = 6 simulated data	*r* ^2^ for *λ* = 100 simulated data
2	0.98	n/a
4	0.98	n/a
6	0.98	0.68
8	0.98	0.72
10	0.98	0.77
20	0.98	0.81
40	0.97	0.83
100	0.94	0.83
200	n/a	0.83
400	n/a	0.80

We list the *r* ^2^ between true and inferred ancestry as a function of the parameter *T* used as input to HAPMIX, for data simulated at *λ* = 6 and *λ* = 100. Results for HAPMIX runs in which the date of admixture was specified correctly are underlined. We did not attempt runs in which *T* differs from the correct date of admixture by a factor of >20.

#### Inference of ancestral populations

We are interested in applying HAPMIX to improve our understanding of ancestral populations contributing to admixture events. To explore the usefulness of the software for this purpose, we analyzed segments of inferred African or inferred European ancestry from our *λ* = 6 and *λ* = 100 simulations to investigate how closely they corresponded to the true ancestral populations used to simulate admixed individuals (see Materials and Methods). We chose to use *F*_ST_, a commonly applied summary statistic, to quantify differences between the inferred and actual ancestral populations. In the *λ* = 6 simulations using Yoruba and French ancestral populations, which closely match the YRI and CEU reference populations, the *F*_ST_ values between segments of inferred ancestry and the corresponding ancestral populations were equal to 0.001, indicating a tight correspondence (Table 3). The *λ* = 100 simulations produced a similarly tight correspondence (Table 3), even though values of local ancestry could only be inferred with moderate accuracy (Figure 3). The correspondence between inferred ancestral segments and true ancestral populations remained reasonably tight even when the true ancestral populations (either Mandenka and Basque, or Yoruba and Druze) were inaccurately modeled by the reference populations (YRI and CEU) used for inference (Table 3). Thus, HAPMIX shows promise for reconstructing ancestral populations that are somewhat different from available reference populations.

**Table 3:** Inference of ancestral populations.

trueAFR	trueEUR	*λ*	*F*_ST_(inferredAFR,trueAFR)	*F*_ST_(inferredEUR,trueEUR)
Yoruba	French	*λ* = 6	0.001	0.001
Yoruba	French	*λ* = 100	0.000	0.003
Mandenka	Basque	*λ* = 6	0.000	0.003
Mandenka	Basque	*λ* = 100	0.001	0.003
Yoruba	Druze	*λ* = 6	0.000	0.006
Yoruba	Druze	*λ* = 100	0.001	0.007

For admixed samples simulated at *λ* = 6 and *λ* = 100 from an ancestral African population (trueAFR) and an ancestral European population (trueEUR), we report the value of *F*_ST_ between segments of African ancestry (inferredAFR) or European ancestry (inferredEUR) inferred by HAPMIX and the true ancestral populations.

Although the correspondence between inferred ancestral segments and true ancestral populations is reasonably tight, it is not perfect, with *F*_ST_ values as large as 0.007 between inferred European segments and the European ancestral population in the Yoruba/Druze simulations (Table 3). Interestingly, the European population with this high *F*_ST_ value contributed only 20% of the ancestry on average in our simulations. We hypothesized that rare erroneous ancestral segments might be having a disproportionate effect on *F*_ST_ estimation for this group, particularly at sites where only a few simulated individuals really had ancestry from the Druze, where errors might dominate. Consistent with this idea, when we restricted our analysis to only positions where we inferred at least 5 chromosomes from the European population, results were considerably more accurate (*F*_ST_ = 0.004 for λ = 100 and *F*_ST_ = 0.003 for λ = 6). Also consistent with this hypothesis, when we repeated the Yoruba/Druze simulations with 50% European ancestry, results were considerably more accurate (0.001 or less for all *F*_ST_ values corresponding to Table 3, for both λ = 6 and λ = 100 and for both European and African segments). Thus, although greater potential for inaccuracy exists in the inference of segments of an ancestral population which on average contributes only a small number of chromosomes to the admixed sample, there is hope of increasing accuracy in this context by appropriate filtering of results.

#### Inference of date of admixture

Our results show that supplying the correct value of the number of generations since admixture to HAPMIX has virtually no impact on the accuracy of inference of local ancestry (Table 2). Nonetheless, inferring the date of admixture remains an important aim for making inferences about history. We tested the effectiveness of HAPMIX in inferring the date of admixture by computing likelihoods at different values of *T*, using data that was simulated at *λ* = 6, *λ* = 20 and *λ* = 100 (see Materials and Methods). The highest likelihoods were obtained at *T* = 6, *T* = 17 and *T* = 75, respectively, with steep likelihood functions leaving little predicted uncertainty in these estimates. Thus, inference of date of admixture is imperfect—with a moderate bias towards underestimation for larger of values of *λ*—but still potentially useful.

We also tried running HAPMIX to infer the date of admixture using data simulated under a double-admixture scenario (*λ* = 6∘100) (see Materials and Methods). This data set violates the model assumption of a single admixture event producing an exponential distribution of ancestry segment lengths. In this simulation, the highest likelihood was obtained at *T* = 45, intermediate between the true admixture times. In the context of multiple admixture events, the HAPMIX date estimate can be loosely interpreted as an estimate of the number of crossover events per unit of genetic distance that have occurred since admixture. We expect this estimate to lie within the time period spanned by the admixture events.

### Analysis of 935 African American samples

We ran HAPMIX on 935 African American samples to obtain local ancestry estimates at each location in the genome (see Materials and Methods). Although the true number of European copies at each locus is unknown, the probabilities produced by HAPMIX provide an estimate of the squared correlation between predicted and true number of European copies (see Materials and Methods). Our estimate was *r* ^2^ = 0.98, which implies that HAPMIX can provide close to full power for admixture mapping of disease genes in African Americans. We also ran the ANCESTRYMAP and LAMP-ANC programs on these data [1],[11] (see Materials and Methods). Discernment of ancestry transitions was much sharper for HAPMIX compared to the other methods, as seen in a plot of number of European copies predicted by each method for an African American sample on chromosome 1 (Figure 2B). This is expected from our results on simulated data (Figure 2A).

In addition to verifying that predictions are accurate on average, it is also important to check that there are no regions of the genome showing systematically inaccurate ancestry predictions. Such regions could produce spurious signals of selection after admixture in scans of control individuals, or spurious admixture association signals in scans of disease cases [13]. Because such scans examine the tail of the observed distribution, even a single region where results are biased could be a serious confounder. With this in mind, we computed the average ancestry across all samples for each locus in the genome, as predicted by either HAPMIX or ANCESTRYMAP, and then searched for unusual deviations. HAPMIX estimates ranged between 16% and 22% European ancestry, and ANCESTRYMAP estimates ranged between 16% and 21%, with a mean of 19% for both methods. These small deviations from the mean are not statistically significant (nominal P-value = 0.001 for the most extreme value over hundreds of independent loci) and can be attributed to sampling variation in the individuals analyzed.

We used HAPMIX to estimate the value of *λ* (the number of generations since admixture) that provided the best fit to the African American data set by computing likelihoods at different values of *T* (see Materials and Methods). We obtained an estimate of *λ* = 7, which matches the value of *λ* = 7.0 inferred by ANCESTRYMAP on the same data, and is similar to the value of *λ* = 6.3 previously inferred by ANCESTRYMAP on other African American data sets [4]. We also used inferred segments of African or European ancestry to estimate *F*_ST_ values between the true ancestral populations of African Americans and the two reference populations used here (YRI and CEU, as well as African and European populations from the HGDP) (see Materials and Methods). We obtained estimates of 0.001 for the *F*_ST_ between the true African ancestral population and YRI, and 0.001 for the *F*_ST_ between the true European ancestral population and CEU. This is consistent with estimates of *F*_ST_ = 0.001 derived from the *τ* parameter inferred by ANCESTRYMAP on the same data (*F*_ST_ = 0.5/*τ*), and consistent with our previous findings that YRI and CEU provide accurate reference populations for admixture analysis of African Americans [4],[25]. Correspondingly, among the HGDP populations the lowest *F*_ST_ to the true African ancestral population was obtained for the Yoruba population (*F*_ST_ = 0.0008). The Bantu South African, Mandenka and Bantu Kenya groups had the next lowest values (*F*_ST_<0.007), and all other African populations showed *F*_ST_>0.035. This supports a West African origin for the African ancestry segments in African Americans, in agreement with historical records. For the European ancestral population, the lowest *F*_ST_ was with French (*F*_ST_ = 0.0013) with Italian, Orcadian, Tuscan, Russian, Basque and Adygei then showing increasing values, but *F*_ST_<0.01 in all cases. This is supportive of a North-West European origin for the majority of the European segments, again agreeing with historical records.

**Figure 5:**
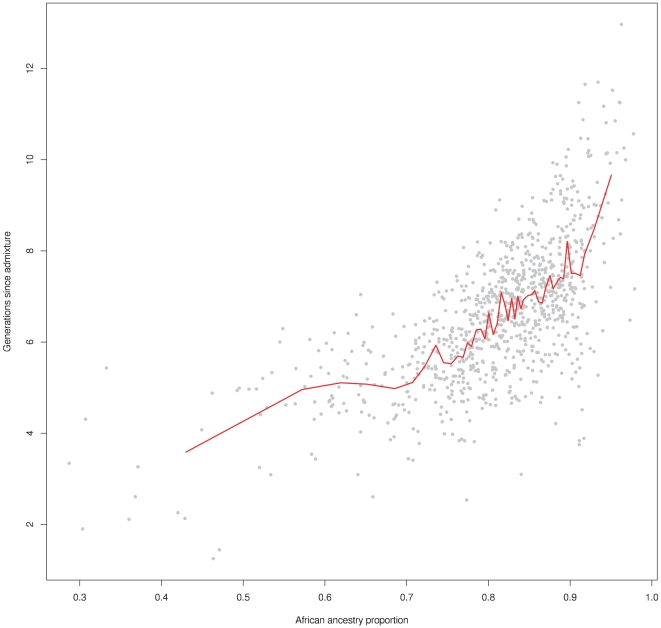
Correlation between ancestry proportion and estimated time since admixture in African Americans. Each grey point shows an estimate of the time λ since admixture corresponding to one of 935 analysed African American individuals (Materials and Methods). The red line shows sliding averages of 20 individuals, binned according to increasing African ancestry proportions.

We sought to investigate whether our precise ancestry inference revealed a correlation between time since admixture and ancestry proportion across individuals. For each individual separately, we estimated a time since admixture (Materials and Methods). The mean estimated time across individuals was 6.62 generations, in close agreement with our *λ* = 7 estimate. However, different individuals showed admixture time estimates ranging from 1.25 generations to 13 generations. Plotting ancestry proportions against these time estimates revealed a striking trend (Figure 5), whereby those individuals carrying higher levels of European ancestry clearly show more recent estimated admixture times. Because individuals with the lowest proportion of European admixture have an estimate admixture time of ∼10 generations, these results demonstrate continuing admixture between Europeans and African Americans over at least 10 generations. Our estimation of admixture time involves rescaling the raw count of ancestry switches, according to the fraction of recombination events since admixture expected to lead to ancestry switches, in a manner dependent on the overall ancestry proportion in the genome (Materials and Methods). We note that individuals with 30%–50% African ancestry show unscaled ancestry switch counts much smaller than for those individuals with 50%–70% African ancestry (p = 0.0007 by Wilcoxon rank sum test), despite the fact that in both groups we expect to observe the same proportion, 42%–50%, of all recombination events, ruling out the idea that the observed trend is simply a consequence of the rescaling.

### Analysis of 29 Mozabite samples

We analyzed 29 HGDP samples from the Mozabite population of North Africa, which has previously been reported to inherit a mixture of both European-related ancestry and ancestry related to sub-Saharan Africans [15],[26] (see Materials and Methods). We therefore continued to use YRI and CEU as input reference populations, to identify segments of sub-Saharan African-related ancestry, and European-related segments. Our analysis aimed to shed light on the origins of the admixing populations, as well as the period in which historical admixture occurred. Runs at a wide range of input *μ*_1_ values all indicated approximately 80% European-related ancestry on average, and thus we fixed the input *μ*_1_ parameter at 80% and ran HAPMIX using a range of input *T* values. The highest likelihood was obtained at *T* = 100 generations. In this run, the average % European-related ancestry of all samples was equal to 78% and the estimated *r* ^2^ between predicted and true number of European copies was 0.79, which is identical to the value we observed in our *λ* = 100 simulations using inaccurate reference populations.

**Figure 6:**
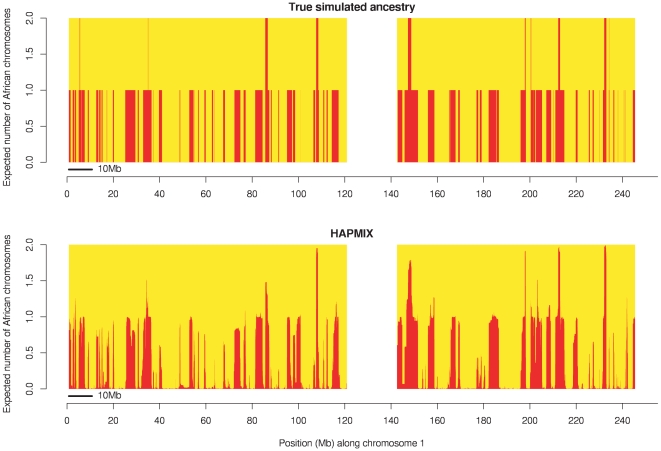
Local ancestry estimates produced by HAPMIX for a simulated anciently admixed sample on chromosome 1, simulated using 80% European and 20% African ancestry, with the admixture occurring 100 generations ago. As in Figure 2, the top plot shows the truth, while the second plot shows the HAPMIX inference. We plot the true number of African chromosomes on chromosome 1 (top plot), together with the number of African copies predicted by HAPMIX (bottom plot).

**Figure 7:**
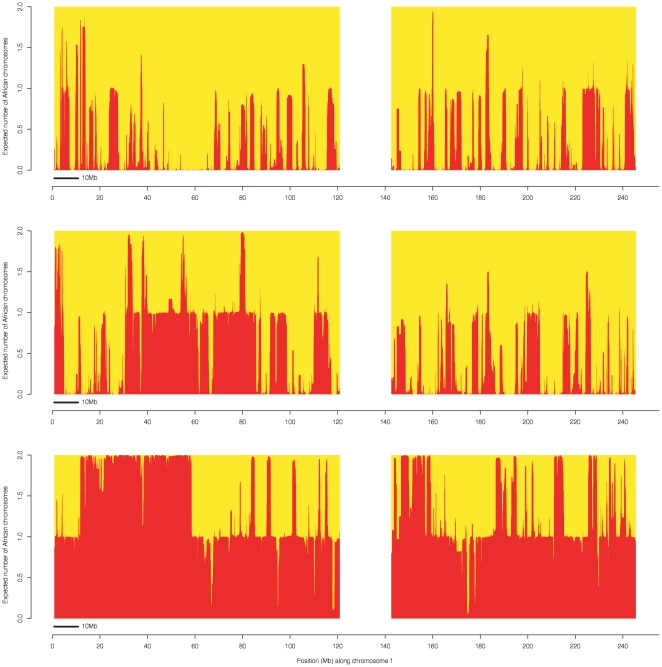
Local ancestry estimates produced by HAPMIX for three real Mozabite individuals on chromosome 1. The plots are constructed as for Figure 5, and show HAPMIX estimates of the number of sub-Saharan African copies across chromosome 1 for three individuals chosen for having different genome-wide African ancestries: 20% (top plot), 29% (middle plot) and 75% (bottom plot). The top plot looks similar to Figure 5, while the much longer segments seen in the two individuals with more African ancestry indicate more recent admixture with sub-Saharan Africans.

We further investigated whether local ancestry inference in Mozabite samples matches our expectations from simulated data by simulating an anciently admixed sample with admixture parameters chosen to be similar to Mozabite. Specifically, we assumed 80% European and 20% African ancestry (French and Yoruba from HGDP) and 100 generations since admixture. HAPMIX results on chromosome 1, along with true ancestry, are displayed in Figure 6. We see that HAPMIX is fairly accurate, but not perfectly accurate, in inferring segments of African ancestry. For comparison, HAPMIX results on chromosome 1 for three different Mozabite individuals are displayed in Figure 7. Results are discussed below, but look generally similar, apart from showing some much larger ancestral segments, to Figure 6.

Different Mozabite individuals within our sample had different estimates of sub-Saharan African ancestry proportions, with a majority at close to 20%, but several individuals having a somewhat higher fraction. Exploration of the causes of this variation (Figure 7) revealed a systematic tendency for those individuals with higher proportions of sub-Saharan African ancestry to have large (tens of megabases) segments in their genome with an African origin. Such large segments are only consistent with admixture within the last 20–30 generations, showing the admixture process has continued into more recent times. In fact, the individual with the highest estimated proportion (75%) of sub-Saharan African ancestry had at least one inferred non-European chromosome throughout virtually their entire genome (Figure 7), consistent with admixture in the last generation, and demonstrating that the admixture process continues today in the Mozabite population. When we restricted our HAPMIX-based dating inference to those two individuals with the highest estimated sub-Saharan African ancestries, we found that the highest likelihood was obtained at 10 generations, much lower than the 100 generations estimated for the combined dataset. In conclusion, the data are most consistent with a model in which individuals from sub-Saharan Africa have been genetically interacting with the Mozabite population as an ongoing process for at least the last 100 generations (∼2800 years) and probably considerably longer, given the underestimation properties of our dating method in simulations, and the likely contribution of recent admixture in producing this estimate. Overall, we were encouraged by the ability of HAPMIX to infer both long and short blocks of distinct continental ancestry in this anciently admixed population.

**Figure 8:**
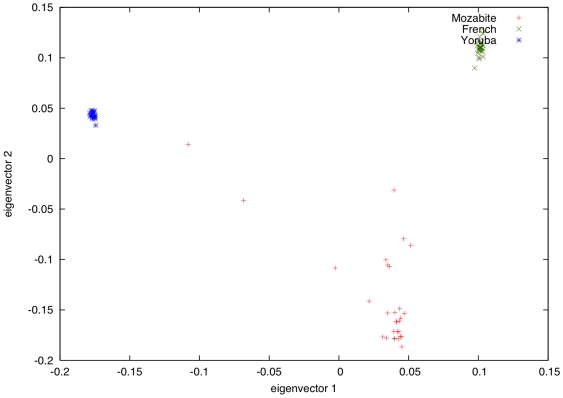
Principal components analysis of Mozabite, French, and Yoruba samples from the HGDP.

Which modern-day populations are most closely related to the founder populations for the Mozabite? Following the promising results of our simulation study, we used inferred segments of African-related or European-related ancestry to estimate *F*_ST_ values between the true ancestral populations of the Mozabite and the two reference populations (YRI and CEU). We obtained estimates of 0.034 for the *F*_ST_ between the true African ancestral population and YRI, and 0.026 for the *F*_ST_ between the true European ancestral population and CEU. Substituting various HGDP Bantu-African and European/West Asian populations for YRI and CEU in the *F*_ST_ computations yielded similar results, with *F*_ST_ values ranging between 0.02 and 0.04. For the African founder population, the West African Mandenka and Yoruba populations, and another HGDP Bantu population, “BantuKenya”, had the smallest *F*_ST_ values (0.034–0.035). For the European-related founder population, the Italians and Tuscans, closely followed by the Palestinians, had the smallest *F*_ST_ values (0.021–0.022), suggesting an origin in South-East Europe or the Middle East. Although care should be taken in interpreting these values, they indicate that the ancestral segments of Mozabite are significantly diverged from extant Bantu-African and European-related populations. To verify this, we ran principal components analysis on the Mozabite samples together with French and Yoruba samples from HGDP, using the EIGENSOFT software [27]. Results are displayed in Figure 8. The first eigenvector indicates, as expected, that the Mozabite samples are intermediate between Europeans and sub-Saharan Africans, consistent with the admixture detected by HAPMIX, and identifying the same two outlier samples with much higher African ancestry. In support of our *F*_ST_ analysis on the ancestry segments, the second eigenvector appears mainly to separate the Mozabite from the other populations, indicating that they are not perfectly modeled as a linear combination of European and African ancestry. Apart from the 2 individuals with much higher African ancestry, the EIGENSOFT plot identifies a further set of 8 Mozabite individuals showing reduced genetic drift (i.e. second eigenvector coefficients), and much more variable ancestry estimates relative to the full set (Figure 8). For these 8 samples, HAPMIX gave a maximum likelihood estimate of 75 generations for the admixture event, again noticeably lower than 100 generations for the full dataset and demonstrating more recent admixture in these individuals. Therefore, we observe a correlation between time since admixture across different individuals, and level of genetic drift relative to modern-day European and African populations. A hypothesis consistent with this finding is that genetic drift has occurred in the Mozabite population itself, during or after admixture, in way that has affected both African and European ancestral segments. Alternatively, the founder populations may have gradually drifted during the thousands of years of admixture that have affected this group.

### Analysis of other HGDP populations

To understand the performance of HAPMIX on real populations with a wider range of histories, we applied the method to 13 different HGDP populations that were of African, Middle Eastern, or European origin. Using YRI and CEU as ancestral populations, HAPMIX inferred that 5 of these populations had greater than 0% and less than 100% European-related ancestry (Table 4). The estimates of European-related ancestry in these 5 populations range from 2%–97%, and the numbers of generations since mixture range from 60–120. The three Middle Eastern populations (Bedouin, Palestinian, and Druze) all show a substantial African-related mixture (3%–9% African-related ancestry). The inferred dates of 60–90 generations correspond to about 2,000–4,000 years ago – contemporaneous with our estimate of the oldest admixture time for the North African Mozabite population – taking into account the fact that HAPMIX systematically underestimate mixture dates by up to 25% for mixtures this old (see simulations above). These results are historically interesting, allowing us to conclude that there is likely to be African ancestry in Middle Eastern populations today that dates to population mixture that occurred in Biblical times. The West African Mandenka population appear to have received ancestry from outside sub-Saharan Africa around the same period or before (120 generations ago). This mixture may not be unexpected, given the Mandenka's geographical location relatively close to the Sahara, and suggests that gene flow across the Sahara has occurred in both directions. Finally, the Middle Eastern results contrast with results for the HGDP European populations, where we consistently estimate the African mixture proportions at close to 0%.

**Table 4:** Results of application of HAPMIX to 13 populations from HGDP that are of African, Middle Eastern or European ancestry, using YRI and CEU as the reference populations.

Population	No. of samples	Estimated percent European ancestry from HAPMIX	Estimated generations since mixture from HAPMIX
Yoruba	21	0%	N/A
Mandenka	21	2%	120
Mozabite	26	77%	100
Bedouin	45	91%	90
Palestinian	41	93%	75
Druze	39	97%	60
Adygei	16	100%	N/A
Basque	24	100%	N/A
French	28	100%	N/A
Italian	12	100%	N/A
Orcadian	14	100%	N/A
Russian	25	100%	N/A
Tuscan	8	100%	N/A

We removed outlier samples from each population using PCA (by making plots like Figure 8, and removing samples that were outliers from the group). For samples with estimated European-related ancestry >0% and <100%, we also inferred the number of generations since mixture.

## Discussion

We have described a method that takes advantage of haplotype information to accurately infer segments of chromosomal ancestry in admixed samples, even in the case of ancient admixture. The method is likely to be useful both for disease mapping in admixed populations and for drawing inferences about human history, as our empirical analyses of samples from African American and HGDP populations have demonstrated. The ability to reconstruct chromosomal segments from ancestral populations that contributed to recent or ancient admixture is a particular advance, as it implies that genetic analyses need not be restricted to extant populations but can also be applied to populations that have only left admixed descendents today [28]. By reconstructing allele frequencies and haplotypes from these populations, extensions of HAPMIX may be able to learn about population relationships as they existed at the time of the Neolithic agricultural migrations or even before. An open question is how far back in time HAPMIX will be able to probe the histories of anciently admixed populations. The simulations of Figure 3 suggest that HAPMIX has power in theory to produce informative estimates of local ancestry even for populations that admixed 400 generations – over 10,000 years ago.

HAPMIX has particularly important applications for disease gene mapping, especially in African Americans where the ancestry estimates are exceedingly accurate and where we have shown that they are not systematically biased. With the accurate estimates of ancestry that emerge from HAPMIX it should be possible to carry out dense case-control association studies with hundreds of thousands of markers, which simultaneously test for admixture association [1]–[3] and case-control association, providing more power to detect disease associations from the data than that can be obtained from either approach alone.

While our analyses show that HAPMIX—because of its explicit use of a population genetic model—has better power to infer locus-specific ancestry than many recent methods, the method also has some limitations in the range of scenarios in which it can be used. For example, it is not currently designed for the analysis of mixtures of more than two ancestral populations, and it requires the use of reference populations. Future directions for extending the HAPMIX method include allowing more than two ancestral populations, using the admixed samples as a pool of reference haplotypes instead of relying on input haplotypes from reference populations, and automating the fitting of model parameters. In addition, although determining the number of generations since admixture with high accuracy is not necessary for effective inference of local ancestry, our results motivate additional work to enable detection of multiple admixture events at different points in time in order to refine the inferences that can be made about human history.

## Supporting Information

Text S1 Supplementary note.
(0.05 MB DOC)

Text S2 Appendix.
(0.10 MB DOC)
