# Supplementary material for: Sensitive Detection of Chromosomal Segments of Distinct Ancestry in Admixed Populations
Source: PLoS Genet. 2009 Jun 19;5(6):e1000519. doi: 10.1371/journal.pgen.1000519 (PMC2689842; doi:10.1371/journal.pgen.1000519)
Supplement: Text S1 — Supplementary note. (0.05 MB DOC) [file pgen.1000519.s001.doc]

Supplementary Note

*Simulations of local ancestry inference for two-way mixtures other than African/European*

We simulated three additional two-way admixtures: (1) African and Chinese, (2) Chinese and European, and (3) Chinese and Japanese. We constructed simulated admixed individuals by applying the previously described procedures (see Materials and Methods, Simulations of local ancestry inference) to 20 diploid samples from each of two corresponding HGDP populations: (1) 80% Yoruba and 20% Han, (2) 80% Han and 20% French, and (3) 80% Han and 20% Japanese. For reference populations, we used the corresponding phased reference panels from HapMap: (1) 120 haploid YRI and 120 haploid CHB+JPT chromosomes, (2) 120 haploid CHB+JPT and 120 haploid CEU chromosomes, and (3) 90 haploid CHB and 88 haploid JPT chromosomes. We used 90 haploid CHB and 30 haploid JPT chromosomes for the 120 haploid CHB+JPT chromosomes, since additional haploid CHB chromosomes were not available; based on *F*_ST_(CHB,JPT)=0.007, this is unlikely to be a major concern (see Results, Simulations of local ancestry inference using inaccurate reference populations). We used a lower number of reference samples in the simulation of Chinese and Japanese, since additional haploid chromosomes were not available. A joint analysis of HGDP and HapMap data indicated that *F*_ST_(Han,CHB)=0.001 and *F*_ST_(Japanese,JPT)=0.001, so that the HGDP populations used to simulate admixed individuals were very similar to the HapMap reference populations used as input to HAPMIX.

HAPMIX and LAMP-ANC results for Yoruba/Han, Han/French and Han/Japanese admixtures simulated at *λ*=6 and *λ*=100 are reported in Table S1. (We did not include the ANCESTRYMAP program in these comparisons, as this would have required a different set of unlinked ancestry-informative markers for each simulation.) In each simulation, HAPMIX outperforms LAMP-ANC. As compared to the Yoruba/French results (Figure 3), results for each method are slightly better for Yoruba/Han, slightly worse for Han/French, and much worse for Han/Japanese, which is consistent with the genetic distances among these pairs of populations. For *λ*=6, using run parameters as described in Materials and Methods, we initially obtained *r*^2^=0.16; corresponding to *r*=0.4, or 60% accuracy for a binary variable, for the Han/Japanese population.

We hypothesized that the low differentiation between groups, *F*_ST_(Han,Japanese)=0.007, might mean the original parameters were inappropriate for this pair of populations. We therefore used single phased haploid JPT and CHB genomes from HapMap, and our E-M procedure (see Materials and Methods), to estimate appropriate parameters for the inference (we fixed the admixture proportions in this procedure so that ancestry was forced to be from the appropriate parental population). This suggested minor modifications to most parameters, but a large increase in the two miscopying rate parameters (Materials and Methods), to 30%. We believe this reflects the closeness of the two populations in this case, so that frequently – around 30% of the time - a Japanese (or Chinese) haplotype segment is more closely ancestrally related to a corresponding segment in the Chinese (Japanese) population sample than to any individual haplotype in the Japanese (Chinese) sample. Rerunning the analysis with the modified parameters (leaving admixture parameters, which we did not fit, unchanged) improved results substantially for *λ*=6 – we obtained *r*^2^=0.38; corresponding to *r*=0.62. Results for *λ*=100 remained poor, and we achieved only *r*^2^=0.06 in this case, even after parameter fitting. While such parameter estimation requires an additional step in the analysis, and appears necessary only in very difficult cases of admixture between highly similar groups, we note that this procedure is applicable for real datasets, because it only requires data from the appropriate reference populations, and not additional knowledge of the admixed group itself.

The cause of the relatively low *r*^2^ values in the Han/Japanese case – although performance is considerably improved relative to LAMP - appears to be mainly the fundamental difficulty of this problem, due to the similarity of the admixing populations. Our results based on simulating admixed individuals using HGDP data were not substantively worse than results for correspondingly simulating admixed individuals based on the HapMap data itself, suggesting that differences between the HGDP Han Chinese and the CHB, or the HGDP Japanese and the JPT, are not key. Further, running principal component analysis on the HGDP data for large numbers of consecutive SNPs indicated that segments containing on the order of tens of thousands of SNPs are needed to reliably separate the two underlying populations (data not shown). It is therefore unclear whether any approach could have high predictive power for identifying admixture chunks in this setting.

*Simulations of local ancestry inference for three-way mixture*

We simulated a three-way admixed population with 60% African, 20% Chinese and 20% European ancestry. We constructed simulated admixed individuals by generalizing the previously described procedures (see Materials and Methods, Simulations of local ancestry inference) to 20 diploid samples from each of three corresponding HGDP populations: Yoruba, Han and French. We sampled lengths of ancestry segments as above, assigned Yoruba, Han or French ancestry to each (haploid) segment using the corresponding probabilities, and merged pairs of haploid admixed individuals to form 20 diploid three-way admixed individuals. We ran HAPMIX in two separate two-way modes, using as HapMap reference populations either (1) 120 haploid YRI and 120 haploid CHB+CEU chromosomes or (2) 120 haploid CHB+JPT and 120 haploid YRI+CEU chromosomes. We used 60 haploid CHB and 60 haploid CEU chromosomes for the 120 haploid CHB+CEU chromosomes, and similarly for YRI+CEU. We multiplied the predictions produced by the two runs to produce probabilities of 2/0/0, 0/2/0, 0/0/2, 1/1/0, 1/0/1, or 0/1/1 copies of African/Chinese/European ancestry—the probability of *a*/*b*/*c* was defined as the probability of *a* copies of African ancestry in run (1) times the probability of *b* copies of Chinese ancestry in run (2)—and normalized the six probabilities to sum to 1. This approach is clearly *ad hoc*, and was developed mainly to investigate the potential of the HAPMIX approach for the 3-way admixture case. We evaluated the normalized combined predictions by computing the squared correlation between the resulting prediction of the number of copies of African ancestry with true number of copies of African ancestry, and similarly for Chinese and European ancestry. We did not include a HAPMIX run using 120 haploid CEU and 120 haploid YRI+CHB chromosomes, because the resulting predictions would be redundant, and would also be less accurate due to the larger genetic distance between YRI and CHB than between any other pair of HapMap populations.

In the three-way mixture simulation, HAPMIX attained *r*^2^ values of 0.98, 0.95 and 0.93 at *λ*=6 and 0.79, 0.65 and 0.58 at *λ*=100 for predicting African, Chinese and European ancestry, respectively. These results are only slightly worse than our two-way mixture simulations (Figure 3 and Table S1), suggesting that running HAPMIX in two separate two-way modes may be an adequate solution to the problem of three-way mixture. However, it may also be valuable to explicitly model three distinct reference populations, an avenue for future work.

Tables

Table S1. Accuracy of HAPMIX and LAMP-ANC predictions for various choices of mixing populations and values of *λ* (the number of generations since admixture). We report squared correlations between predicted and true ancestry based on an analysis of 20 admixed individuals that were simulated at the specified value *λ* using average genome-wide ancestry proportions of 80% from POP1 and 20% ancestry from POP2 and modeled using reference populations from HapMap. For the Han-Japanese admixtures, reported HAPMIX accuracies refer to runs using parameters inferred using the EM algorithm (see Supplementary Note text) with accuracies attained using identical parameters to those employed for the other simulations shown in parentheses.

| POP1 | POP2 | *λ* | HAPMIX | LAMP-ANC |
| --- | --- | --- | --- | --- |
| Yoruba | Han | *λ*=6 | 0.99 | 0.85 |
| Yoruba | Han | *λ*=100 | 0.86 | 0.54 |
| Han | French | *λ*=6 | 0.95 | 0.81 |
| Han | French | *λ*=100 | 0.67 | 0.28 |
| Han | Japanese | *λ*=6 | 0.38 (0.16) | 0.13 |
| Han | Japanese | *λ*=100 | 0.06 (0.05) | 0.01 |
