# Supplementary material for: Sensitive Detection of Chromosomal Segments of Distinct Ancestry in Admixed Populations
Source: PLoS Genet. 2009 Jun 19;5(6):e1000519. doi: 10.1371/journal.pgen.1000519 (PMC2689842; doi:10.1371/journal.pgen.1000519)
Supplement: Text S2 — Appendix. (0.10 MB DOC) [file pgen.1000519.s002.doc]

Appendix

*Inferring parameters in the model used by HAPMIX via the EM algorithm*

The model as defined in Materials and Methods has a non-trivial number of parameters. Specifically, there are 3 mutation parameters, 2 recombination scaling parameters, 2 miscopying parameters, and admixture times and genomic proportions for each chromosome being analyzed. Although in many scenarios reasonable values for these parameters can be pre-specified (main text), we have also implemented an EM algorithm approach to estimate these parameters directly via maximum-likelihood. Our implementation currently assumes that haplotypic phase is known, and can estimate parameters for multiple individuals either separately or jointly, as well as allowing estimation of any subset of the 9 parameters. It would be simple to extend the algorithm to the case of unknown “phase”, but because the corresponding HMM has an increased number of states in this case, in some settings full parameter estimation is likely to be computationally infeasible for unphased data.

In order to develop our EM algorithm, we view the underlying state transition process in our HMM as occurring in continuous space; i.e. we model the genome as continuous, with positions given in terms of the genetic, not physical map, and emissions (i.e. observed genotypes) occurring at certain positions along this space. Conceptually, we can imagine complete data as being the collection of hidden states at every position along the genome, the positions of ancestral recombination events (even if these do not change the underlying hidden state), and finally the genotype data itself for the haplotype of interest. In order to simplify our procedure, it is convenient to also add information about whether at recombination points, the recombination occurred more recently than the time of admixture, as part of the ancestry switching process which is occurring at rate *T*, or anciently as part of the within-population process at rate *ρ*_1_ or *ρ*_2_.

Recall that the underlying hidden states are of the form (*i,j,k*) where *i*=1 or 2 represents ancestry drawn from population 1 or population 2, *j*=1 or 2 records the population the chromosome copies from at a given position, and *k* represents the individual used for copying. Suppose that the total length of the region of interest is *L* in genetic distance. Suppose further that a length *l*_1_ is spent in ancestry state 1, while *l*_2_ is spent in ancestry state 2, where *l*_1_+*l*_2_= *L*.

For each pair (*i,j*) and (*l,m*) of ancestry and copy population states let $n_{_{i,j,l,m}}^{a}$denote the total number of switches from state pair (*i,j*) to state pair (*l,m*) which occur as a result of ancestry switching, and $n_{_{i,j,l,m}}^{r}$ be the number which occur as a result of ancient within-population recombination switches. Further define indicator variables $I_{i,j}$=1 if at the first position along the chromosome the underlying hidden state is state (*i,j*) and 0 otherwise. Finally, denote indicator variables $J_{i,j,k}(s)$=1 if at a genotyped site at position *s*, the underlying state is (*i,j,k*) and there is a mutation event (i.e. individual k in population j differs from the observed haplotype at position s, and is 0 otherwise, and $K_{i,j,k}(s)$is the corresponding indicator variables, which is equal to 1 where there is not a mutation event. We denote sums of these variables using a dot notation – so for example $n_{i,\bullet,l,m}=n_{i,1,l,m}+n_{i,2,l,m}$.

It is straightforward to see that the log-likelihood of this complete data is given by

Given a current parameter set, the E-M algorithm proceeds by choosing updated parameters which maximize the expected log-likelihood (where the expectation is taken conditional on the current parameter set), and iterating this process to convergence, to at least a local maximum of the likelihood.

This leads to the following updated parameters given a current set of parameters $(\theta_{1}^{n},\theta_{2}^{n},\theta_{3}^{n},\rho_{1}^{n},\rho_{2}^{n},\mu_{_{1}}^{n},T^{n},p_{_{1}}^{n},p_{2}^{n})$ at step *n* of the algorithm:

where, for example, $E^{n}(l{}_{1})$denotes the posterior expectation of *l*_1_ calculated conditional on the parameters at step *n* of the E-M algorithm. Although we do not give complete details here, the above expressions for the update step can be calculated easily, and efficiently, using the HMM structure of our model. Each update step requires a single forward-backward pass through the data.

When combining information for multiple individuals, or multiple chromosomes in one individual, we can simply add the log-likelihoods. Further, because the log-likelihood expression separates in terms of the different parameters, it is trivial to modify the algorithm to keep any subset of these parameters fixed, and update only the others, if prior information is available. Both these options are implemented in HAPMIX.

*Additional speedup for diploid genotype data*

A major challenge for analyzing genome-wide datasets with multiple individuals and unphased genotype data is computational speed. Our algorithm requires parental data for each reference population, introducing additional hidden states compared to software that analyses single populations [1,2]. These issues are potentially serious when the challenge is to analyze data from thousands of individuals as is expected to be necessary for disease gene mapping.

In order to address these issues, we have introduced a modification to the underlying HMM model that results in a reduction of the number of hidden states at each position. Our approach is related to others that have been developed in the single-population setting [3,1] but is somewhat simpler. Our aim here is not to improve the accuracy of population inference (in practice, using the approximation typically has very little impact on the quality of such inference), but purely to increase the speed of the code (we found that it can increase computation speed 20-fold or more; results not shown). The new model has a single user-set parameter *d*, which defines the extent to which the underlying HMM is approximated. As this parameter becomes large, the algorithm converges to the HMM described in Materials and Methods , at the cost of increased computation time. One advantage of our modification is that it remains model-based, allowing interpretation of parameters, particularly as the model converges to our original HMM for large *d*. In addition, the algorithm is deterministic, avoiding potential convergence issues.

We describe our modification for the case where our admixed data is in the form of haplotypes. The extension to the genotype case proceeds exactly as for the original algorithm. The parameter *d* defines a distance in genetic units (e.g. 0.1cM). The original phased parental samples *P*_1_, *P*_2_ contain *n*_1_, *n*_2_ individuals respectively. Suppose we are considering states at site *s*. In the original formulation, the state at site s is a triplet (*i,j,k*) where *i* and *j* represent the underlying and copy populations, and *k* the individual copied from in population *j*, at this site. The modified algorithm begins by defining haplotype classes at site *s*, defined based on the variation patterns at all sites within distance *d* of this specific site. This defines a total of $n_{1}^{s}\leq n_{1}$, $n_{2}^{s}\leq n_{2}$ classes for *P*_1_, *P*_2_ respectively. In the new formulation, a state at site *s* is again of the form (*i,j,k*) but now $1\leq k\leq n_{j}^{s}$ and the “*k*” state now corresponds to the *haplotype* type of the individual copied at site *s*. The reduction in number of hidden states we must consider comes from the fact that there are potentially many fewer haplotypes than parental individuals, particularly if *d* is chosen to be relatively small.

In the original formulation, the prior probability of every choice of *k* is simply taken uniformly on possibilities. In the new formulation, some haplotypes will be more common than others. Therefore, if haplotype *k* occurs $T_{j}^{k}(s)$ times in total in the reference population *j* sample, it is given a prior weight of $T_{j}^{k}(s)/n_{j}^{s}$. Further, we now need to account for the fact that as the offspring hypothetically moves along a single “parent” individual whose haplotype is being copied, then even without recombination occurring the underlying haplotypic state label of this individual may now change between sites *s* and (*s*+1), because haplotypes at site (*s*+1) are redefined, independently of those at site *s*. Therefore, we also require a “no recombination” transition probability between the newly defined parental states. If switches from haplotype *k* at site *s* to haplotype *l* at site (*s*+1) in the population *j* sample occur $T_{j}^{kl}(s)$ times in total, the corresponding forward transition probability is naturally set to $T_{j}^{kl}(s)/T_{j}^{k}(s)$. We can formally write down the new transition probabilities as follows:

Finally, we consider emission probabilities at site *s*. We note that because the haplotype definition at this site includes the type at site *s* itself, each new state is associated with a unique type at position *s*, and this is used in calculating emission probabilities, exactly as in the original approach. We can now go on to estimate posterior probabilities of hidden states, and underlying populations, by adapting our previous algorithm. As before, algorithmic speedups can be implemented, so that the run time is linear in the number of hidden states.

As the “haplotype length” *d* becomes large, all haplotypes in the parental sample will become unique everywhere, and in this case, it is easy to verify that the transition probabilities above revert to those defined for the unapproximated model.

We feel that by choosing a set *genetic* distance *d*, rather than a physical distance, our haplotype definition is likely to gain some helpful properties. First, in higher LD regions, more sites on either side of *s* will be considered, enabling utilization of long range LD present at such positions. To avoid very high recombination rates resulting in very short haplotypes at some positions, in our implementation we in fact always include at least 2 segregating sites on each side of each site *s*. Second, by considering local haplotype diversity, the approach will have fewer states in less diverse regions, where in some sense variation patterns are “simple”, while spending more effort in regions with complex variation patterns. The drawback of the approach is that it is not lossless - all potential parents in the original sample who have the same haplotype for a distance d on either side of site s are forced to have an identical weight. This means we effectively ignore some potential information from longer range LD, although the transition process is expected to capture some of this. If *d* is set too small, this failure to account for long range LD can affect the quality of inference. However, in practice we have found that by choosing *d* to be suitably large – a value of 0.05cM is used throughout this paper – the loss of information is very slight, although unsurprisingly in simulated data performance appears to monotonically improve as *d* increases. The approximation is only implemented for genotype data, because in the phase-known setting, our original model allows for rapid computation.

References

1. Browning SR, Browning BL (2007) Rapid and accurate haplotype phasing and missing-data inference for whole-genome association studies by use of localized haplotype clustering. Am J Hum Genet 81: 1084-97.
2. Marchini J, Howie B, Myers S, McVean G, Donnelly P (2007) A new multipoint method for genome-wide association studies by imputation of genotypes. Nat Genet 39: 906-13.
3. Scheet P, Stephens M (2006) A fast and flexible statistical model for large-scale population genotype data: applications to inferring missing genotypes and haplotypic phase. Am J Hum Genet 78: 629-44.
